# Assessing Primary Care Physicians’ Readiness for AI-Based Adaptive Learning: Perceptions, Barriers, and Learning Needs in Northern Saudi Arabia

**DOI:** 10.3390/healthcare14070865

**Published:** 2026-03-27

**Authors:** Bashayer Farhan ALruwaili, Asma Naeem Alruwili, Asma Muaysh Alruwaili, Huriyyah Saad Alruwaili, Norah Awadh Almutairi, Taif Talal Alruwaili, Buruj Tariq Alsirhani, Ashokkumar Thirunavukkarasu, Hajar Ismail AL-Ruwaili

**Affiliations:** 1 Department of Family and Community Medicine, College of Medicine, Jouf University, Sakaka 72388, Saudi Arabia; ashokkumar@ju.edu.sa; 2 Department of Family Medicine, Public Health Authority (Waqaya), Northern Border Region, Arar 73311, Saudi Arabia; asnalruwili@moh.gov.sa; 3 College of Medicine, Jouf University, Sakaka 72388, Saudi Arabia; asmaalrwaili24@gmail.com (A.M.A.); huriyyahalruwaili@gmail.com (H.S.A.); norahalmu820@gmail.com (N.A.A.); ttayf6644@gmail.com (T.T.A.); brooj20033@gmail.com (B.T.A.); 4 Department of Public Health, Ministry of Health, Sakaka 72341, Saudi Arabia; haialruwaili@moh.gov.sa

**Keywords:** primary healthcare, family medicine, AI-based adaptive learning, perceptions, barriers, Saudi Arabia

## Abstract

**Background and objectives:** Artificial intelligence (AI)-based adaptive learning has the potential to strengthen clinical decision-making and enhance quality of care at primary health centers. The present study assessed the perceptions, barriers, and learning needs involved in AI-based adaptive learning among primary care physicians in Northern Saudi Arabia. **Methods:** We used a cross-sectional study design to obtain data from 285 primary care physicians of different cadres working in various primary health centers. A validated data collection tool was used to measure three domains: perceptions, barriers, and learning needs. A multivariable analysis was carried out to identify the factors associated with these three domains. **Results:** Among the studied participants, low perceptions were observed in 55.1% of physicians; they were higher among those aged >40 years (*p* = 0.019) and non-Saudi nationals (*p* = 0.003). High barriers were reported by 42.5% of respondents, and this was higher among those aged >40 years (*p* = 0.031). Learning needs were higher among non-Saudi nationals (*p* = 0.017) and those with >10 years of experience (*p* = 0.007). The perception and learning need scores were positively correlated, and barrier scores were negatively correlated with the other two domains (*p* < 0.001). **Conclusions:** The authorities concerned may consider implementing targeted measures for AI-based adaptive learning. Moreover, efforts should be made to reduce the barriers to AI-based adaptive learning at all levels. These measures could strengthen primary care practice and enhance patient care.

## 1. Introduction

Artificial intelligence (AI) is reshaping healthcare delivery and administrative management through its ability to run advanced diagnostic programs, organize clinical support tools, and streamline operational work processes [1,2]. The healthcare field utilizes AI to enhance educational experiences, create personalized instruction, and sustain career development for medical professionals. Medical students, physicians, and educators manage massive challenges, from expanding medical knowledge and dynamic clinical guidelines to intricate patient care structures [3,4]. Pre-existing didactic teaching methods fail to fulfill the learning requirements of students who need personalized educational approaches in rapidly changing clinical environments. This situation has revealed the urgent necessity for new, flexible educational methods focused on learner development [4,5]. The educational requirements can be satisfied through AI-based adaptive learning platforms that deliver interactive, personalized instruction based on user-specific data patterns [6,7].

The adaptive learning platforms guide learners through personalized education paths so that time is used efficiently, focusing on the aspects that deliver the greatest learning potential [7,8,9]. The educational systems utilize real-time feedback, clinical simulation, and assessment analytics to deliver an engaging learning experience. The value of adaptive learning systems intensifies within clinical education since physicians require capabilities for theoretical understanding and scenario-based application of knowledge in complex medical practices [4,10].

The adaptive learning technology supported by AI is consistent with a focus on lifelong learning that healthcare professionals need due to rapidly developing knowledge bases [11]. Family medicine practitioners handle extensive acute and chronic conditions for patients of all ages. Through adaptive learning, they can access educational content that matches the real-world aspects of their clinical practice. This system enables physicians to conduct immediate knowledge updates and patient scenario-based customization of their learning activities. Educational institutions worldwide have started implementing adaptive learning platforms that improve conventional teaching programs while building residency training systems and delivering ongoing medical education to their students. Previous studies observed that these platforms enhance student learning outcomes, satisfaction levels, and mastery of medical skills [12,13,14].

Medical professionals’ clinical competence depends heavily on Continuing Medical Education (CME), as physicians serving family medicine patients need to manage various healthcare issues among different demographic groups. Family physicians need to deliver complete personalized care, from disease prevention to holistic treatment [15,16]. Lectures, workshops, and online modules that comprise traditional CME delivery methods present standardized content without catering to the specific learning needs of individual healthcare providers. Currently, available learning methods fail to meet the needs of healthcare providers, so new individualized learning approaches are essential [17,18]. AI-based adaptive learning presents an adaptable solution by personalizing learning requirements as per individual proficiency, pace, and specialization. Combining technology with learning methods benefits family medicine practitioners through more efficient continuing medical education, producing higher-impact results [8,19,20].

Saudi Arabia is currently undergoing major healthcare system reform through Saudi Vision 2030, focusing on digital progress, workforce development, and higher accessibility to quality healthcare services. The transformation strategy includes utilizing technology for healthcare education, together with continuous professional development programs [21]. Family medicine serves as a vital foundation for the country’s existing primary healthcare reform [22,23]. Healthcare infrastructure and training resources remain more limited in northern Saudi Arabia than in its major urban centers, thus creating obstacles for primary care physicians to access specialized CME opportunities. Nonetheless, devices powered by AI adaptive learning platforms can overcome these knowledge gaps by delivering personalized, flexible education solutions [4,8,12]. Any successful deployment of these tools demands complete knowledge about the specific situation in which they are used, particularly with regard to family physicians’ readiness, perceptions, and learning requirements. Implementing future digital learning programs will succeed by understanding local cultural needs, healthcare priorities, and professional practice demands.

AI-based adaptive learning has the potential to strengthen clinical decision-making and guideline adherence, in turn enhancing the quality of care at primary health centers (PHCs). However, successful implementation of this method requires understanding the local readiness level and existing needs, as well as potential implementation barriers. Assessing this possibility within family medicine practice is a critical contemporary issue. Some authors have explored these aspects among physicians from different specialties and medical students [24,25,26]. Readiness to adopt technology-enhanced learning in healthcare may be understood as a multidimensional construct shaped by physicians’ perceptions, perceived barriers, and learning needs. These domains are interrelated and together influence preparedness to engage with AI-based adaptive learning [27,28,29]. The focus on Northern Saudi Arabia is relevant within the Kingdom’s broader digital health and AI transformation agenda under Saudi Vision 2030. National initiatives, including the Health Sector Transformation Program, the Ministry of Health’s digital transformation efforts, and the National Strategy for Data and AI, highlight the growing importance of digital and AI-enabled healthcare systems [21,30,31]. In this context, assessing primary care physicians’ readiness in Northern Saudi Arabia is important, especially given the limited regional evidence. Therefore, this research assessed primary care physicians’ readiness for AI-based adaptive learning in Northern Saudi Arabia by examining three interrelated domains: perceptions, perceived barriers, and learning needs. The findings are expected to inform health authorities about options regarding the implementation of strategies that strengthen primary care practice and ultimately enhance patient care.

## 2. Materials and Methods

### 2.1. Study Design

The present study utilized a cross-sectional study design and was conducted from August 2025 to December 2025.

### 2.2. Study Setting

This study was performed in two provinces of northern Saudi Arabia, namely, Aljouf and the northern border. We included primary care physicians, defined as physicians providing direct primary care services in PHC settings, at all levels (residents, specialists, and consultants), both Saudi and non-Saudi nationals, working in PHCs for a minimum duration of 6 months. We excluded unwilling participants, those who were on leave, and the family medicine doctors working at specialized hospitals.

### 2.3. Sample Size Estimation

We used an online sample size calculator based on Cochran’s formula to estimate the required number of primary care physicians for our study [32]. We used a 95% confidence level and a margin of error set at 0.05, and estimated the proportion of the attribute present in the population (assumed to be 50% to maximize variability and obtain the highest number of physicians). Furthermore, the sample size was adjusted to the total number of primary care physicians (1091) working in the PHCs of these two provinces. After applying all these values, we finalized the minimum required number of participants as 285.

### 2.4. Sampling Method

The present study used the non-probability convenience sampling method to recruit participants from different cadres and practice settings in Northern Saudi Arabia. Participants were identified and recruited through coordination with local Ministry of Health directorates, residency program coordinators, and primary healthcare administrators. This approach was considered appropriate for efficiently accessing eligible physicians across multiple PHC settings and professional cadres within the available study period. The willing participants were approached according to their free time during working hours. Even though we used convenience sampling, efforts were made to ensure proper representation across regions and physician cadres.

### 2.5. Data Collection Steps

The research was conducted in accordance with the Helsinki Declaration and local guidelines. We initiated data collection after obtaining approval from the IRB, Jouf University (approval no. 7575, dated 1 June 2025), and other concerned authorities. Data collectors conducted on-site recruitment and asked eligible physicians to complete the questionnaire; all eligible physicians who were available and willing at the time of the visit were enrolled after confirming eligibility and providing informed consent. After a brief standardized explanation of AI-based adaptive learning as an educational approach that uses artificial intelligence to adjust learning content, pace, feedback, and learning pathways according to individual learners’ needs, performance, and progress, along with the study objectives and participants’ roles [10,33]. We asked the selected primary care physicians to complete the data collection form (Google Form) on their personal devices.

The questionnaire items were developed based on relevant literature and were refined through expert review before pilot testing and psychometric evaluation [34,35,36]. We conducted a pilot study among 31 primary care physicians from different cadres, and feedback indicated that the tool was easy to comprehend and took about 10 min to use. The data collection form consisted of five sections. The first section asked about the physician’s background details. The second section inquired about current CME practice. The third section asked participants about their perceptions of AI-based adaptive learning in clinical practice and CME. The fourth section asked about participants’ barriers to adopting AI in clinical practice and CME. The third and fourth sections consisted of 7 questions each, and participants responded on a 5-point Likert scale ranging from strongly agree (5) to strongly disagree (1). Responses in the perception domain were scored on a 5-point Likert scale, and negatively worded items (items 4, 6, and 7) were reverse-scored prior to calculating the composite perception score to ensure that higher total scores consistently represented more favorable perceptions toward AI-based adaptive learning. The final section asked about primary care physicians’ learning needs in 8 items, with responses ranging from not needed (1 point) to highly needed (5 points). Finally, we computed all scores for further analysis, as discussed in the next section. The Cronbach’s alpha values for the perceptions, barriers, and learning needs were 0.77, 0.81, and 0.83, respectively. Exploratory factor analysis (EFA) supported the construct validity of the instrument, with all items demonstrating factors loading above 0.60. Further details of the EFA are provided in the Supplementary Material (Supplementary File S1).

### 2.6. Data Analysis

We used the Statistical Package for the Social Sciences (SPSS), version 21.0 (IBM Corp., Armonk, NY, USA) to export data from Excel downloaded from Google Forms and to analyze the data. The descriptive data are presented as numbers and proportions for categorical variables and as mean, standard deviation, median, and interquartile range (IQR) for continuous variables. After assessing the normality assumption, we found that the data in the present study were skewed. Therefore, we applied Spearman’s rank correlation test to assess the strength and direction of the correlation between perceptions, barriers, and learning need scores. Given the skewed data, we used multivariable analysis with binomial logistic regression (enter method). The independent variables included age group, gender, nationality, highest qualification, current position, work experience, and use of digital tools for clinical learning. Multicollinearity among the independent variables was assessed before model fitting, and no problematic multicollinearity was detected. In this method, we categorized each domain using a median split into low (≤median) and high (>median). The corresponding median values were 24 for perceptions, 21 for barriers, and 31 for learning needs. This method is commonly used in scientific research while performing multivariate analysis for skewed data [37,38]. A *p*-value of less than 0.05 was considered a statistically significant value.

## 3. Results

During the data collection period, we approached 347 eligible participants to obtain a minimum required sample of 285 participants (response rate: 82.1%). A flow diagram of participant recruitment until 82.1% is included as a Supplementary File (Figure S1). Of the studied participants, the majority of physicians belonged to the 31 to 40 age group (mean ± SD = 35.29 ± 7.79), and were female (54.0%), Saudi nationals (72.3%), had Saudi board examination certificate as the highest qualification (49.5%), were specialists (34.0%), and had work experience of 5 to 10 years (37.9%). More than three quarters (78.2%) used digital tools for clinical reasoning in their practice at primary care settings (Table 1).

Most participants reported engaging in CME at least every 2–3 months (38.9%) or monthly (27.7%), and nearly three quarters had attended CME related to digital health/AI/e-learning (73.7%). Online/webinar-based CME was the most preferred format (88.4%), followed by mobile-based apps (72.3%) and in-person sessions (69.5%), with professional development (40.4%) and interest in the topic (20.7%) being the leading motivations (Table 2).

Supplementary Table S1 presents the primary care physicians’ responses regarding perceptions toward AI-based adaptive learning. The strongest agreement was found for the statement “I believe AI-based learning tools can improve the effectiveness of CME for primary care” (42.5%), followed by the statement “Personalized learning tools would help me better identify and address my clinical knowledge gaps” (40.7%).

Supplementary Table S2 presents the primary care physicians’ responses to barriers to adopting AI-based adaptive learning. Of the 285 participants, most of the participants either agreed (36.8%) or strongly agreed (11.2%) with the statement “I am concerned that over-reliance on AI tools might reduce my critical thinking over time.” The lowest proportion of the participants either agreed (22.1%) or strongly agreed (7.0%) with the statement “I am not confident in using digital tools for education”, followed by the statement “My institution does not currently support or promote AI-based learning platforms [agree: 21.4%, strongly agree: 7.7%].”

Supplementary Table S3 shows the learning needs for AI-based adaptive learning implementation among the participants. Of the studied participants, the highest proportion indicated a need or high need for preventive care and screening guidelines across age groups (needed: 41.1%; highly needed: 22.8%), followed by mental health assessment and management in primary care (needed: 37.2%; highly needed: 25.3%) and interpretation of evidence-based guidelines and research for practice (needed: 36.1%; highly needed: 25.6%).

The descriptive statistics of the three domains are shown in Table 3. The median (IQR) scores were 24 (6) for perceptions, 21 (8) for barriers, and 31 (11) for learning needs. The mean ± SD scores were 24.47 ± 4.50, 21.61 ± 5.81, and 29.11 ± 7.68, respectively, for the same domains.

Spearman’s correlation (non-parametric) test results are shown in Table 4. The perception score was moderately negatively correlated with barriers (Spearman’s correlation coefficient [ρ] = −0.490, *p* < 0.001) and moderately positively correlated with learning needs (ρ = 0.539, *p* < 0.001). The learning needs score was moderately negatively correlated with barriers (ρ = −0.402, *p* < 0.001).

Of the participants studied, 55.1% had low levels of perception towards AI-based adaptive learning. Regarding factors associated with the perception of AI-based adaptive learning, the lower levels of perception were significantly higher among those aged more than 40 years old (ref: up to 30 years, adjusted odds ratio [AOR] = 2.14, 95% confidence intervals [CI] = 1.32–3.08, *p* = 0.019), non-Saudi nationals (ref: Saudi, AOR = 2.67, 95% CI = 1.24–4.19, *p* = 0.003), and those who had work experience of more than 10 years (ref: Up to 5 years, AOR = 2.93, 95% CI = 1.75–4.14, *p* = 0.001). Those who used digital tools for clinical learning had lower odds of having low perceptions toward AI-based adaptive learning compared with those who did not use digital tools (AOR = 0.72, 95% CI: 0.61–0.93, *p* = 0.038) (Table 5).

Table 6 depicts the factors associated with AI-based adaptive learning. High levels of barriers were observed among 42.5% of the physicians. Regarding factors associated with the barriers to adopting AI-based adaptive learning, the higher levels of barriers were significantly higher among those aged more than 40 years old (ref: up to 30 years, AOR = 2.59, 95% CI = 1.37–3.74, *p* = 0.031) and those who had work experience of more than 10 years (ref: Up to 5 years, AOR = 1.56, 95% CI = 1.19–1.86, *p* = 0.020).

Factors associated with learning needs for AI-based adaptive learning are shown in Table 7. The learning needs were significantly higher among non-Saudi nationals (ref: Saudi, AOR = 1.65, 95% CI = 1.06–2.38, *p* = 0.017), those who had MD or equivalent qualification (ref: MBBS, AOR = 1.70, 95% CI = 1.29–2.86, *p* = 0.008), those who had work experience of 5 to 10 years (ref: Up to 5 years, AOR = 1.77, 95% CI = 1.14–2.46, *p* = 0.035), and those who had work experience of more than 10 years (ref: Up to 5 years, AOR = 2.65, 95% CI = 1.38–4.01, *p* = 0.007).

## 4. Discussion

Primary healthcare systems increasingly rely on digital solutions to strengthen workforce capability and quality of care. In addition, understanding whether PHC physicians are prepared to adopt AI-based adaptive learning is essential for successful implementation. Therefore, this study examined northern Saudi primary care physicians’ readiness by examining perceptions, perceived barriers, and learning needs related to AI-based adaptive learning. These results can also be interpreted in terms of TAM and UTAUT, where adoption is determined by perceived usefulness, effort expectancy, and facilitating conditions. In this respect, the generally favorable perceptions in our study might be due to the expected value, and the presented barriers and learning needs indicate that readiness for AI-based adaptive learning is not merely dependent on attitudes, but also on confidence, institutional support, and practical preparedness [39,40].

We found that more than three quarters of the participants already used digital tools for clinical reasoning in their practice at primary care settings. This finding suggests that PHC physicians are not “technology-naïve,” and it would be easy to build on AI-adaptive learning based on existing habits. Previous studies on the use of digital tools yielded varying results. For example, Al-Ghamdi S reported that 53% of their participants used digital tools at least once a day [41]. In contrast, Alqahtani SS et al. reported that about 87% of participants used digital resources at their workplaces [42].

Regarding participants’ CME practices, the data suggest that primary care physicians in the PHC setting are already engaged in continuing professional development and are generally receptive to technology-based learning. The majority of participants reported regularly engaging in CME, and almost three-quarters attended CME on digital health/AI/e-learning. These are critical bases for increasing exposure to technology-focused education and potentially mitigating resistance to AI-assisted learning methods [43,44]. Even though approximately 70% of the participants preferred traditional CME methods, they are one of the choices in our study, and the high demand for online/web-based formats and mobile-based learning also indicates the practicality of providing adaptive learning in formats already popular among physicians. In contrast, Al-Sheikhly D et al., in their web-based survey disseminated among primary care physicians in Qatar, stated that over 90% of the study participants preferred traditional lectures, workshops, case-based sessions, small group, and online self-paced learning [45]. All these trends together offer an effective basis for working with PHCs through time-efficient learning designs that can accommodate high-workload settings. The authorities concerned may consider hybrid approaches (traditional and online) to better meet their learning needs [46].

Physicians in our study generally responded to AI-based learning with more agreement and a positive perception. In Saudi Arabia and globally, some studies have reported similar and contrasting findings to the present study participants’ perception. For instance, a 2025 survey found that the majority of Pakistani medical students had a positive attitude toward AI as an educational tool [47]. Likewise, most Saudi physicians in a recent survey trusted AI for clinical decision support and believed it increased efficiency [48]. In Bahrain, practicing physicians were also largely positive about AI in medicine, viewing it as beneficial even though many worried about job impacts [49]. Furthermore, a recent systematic review (2025) by Abdulazeem HM et al. echoed our findings: that many clinicians express caution, voicing concerns about patient safety, privacy, or job security [44].

In the present study, we found that the lower-level favorable perceptions (low perceptions) were significantly higher among those aged 40 or older and those with more than 10 years of work experience. Our findings are supported by some studies [50,51], including a recent (2026) online study conducted by Kim B et al. among primary care physicians in the USA. In their study, younger physicians were more likely to accept and endorse AI tools [52]. Possible reasons for the present study’s findings include the fact that older/more experienced physicians often have more established clinical routines. The lower perception scores and higher perceived barriers observed among physicians aged >40 years may partly reflect generational differences in digital literacy and familiarity with technology-enabled learning. This suggests the need for age-sensitive orientation and practical support during implementation [29,53]. However, in contrast to our study, a mixed-methods physician survey by Heinrichs H et al. reported that age did not influence attitudes, with attitudes shaped more by engagement/familiarity and role-related experience [54]. Similar to the earlier studies, we found that physicians already using digital learning tools were more open to AI-based adaptive learning [48,52]. This pattern is also consistent with broader digital-health adoption literature, which suggests that engagement with technology-based services is shaped by perceived value, usability, and user confidence [55].

The higher levels of less favorable perceptions among the expatriate physicians could be due to communication demands, variable institutional support, and differing access to locally endorsed professional development pathways. Some studies from Saudi Arabia have demonstrated expatriate challenges, including intercultural competencies and challenges faced by expatriate professionals [56,57]. Based on these results, health leaders can consider adopting more specific tactics, including greater support for older and more experienced physicians, as well as expatriate physicians, to increase confidence and acceptance of AI-based adaptive learning. This may enhance guideline-appropriate care at the primary healthcare level.

The present study participants’ barrier item responses indicate fear that over-reliance on AI could weaken critical thinking and clinical autonomy. This finding is similar to findings from other studies among physicians. In general, clinicians often support AI in principle but remain cautious about dependency, errors, transparency, and accountability [28,54]. The observed higher perceived barriers among older physicians could be similar to the perception contexts. Therefore, the PHC authorities should consider providing periodic sessions on the implementation of a risk-mitigation approach, including a safe approach, and on critical appraisal for older/more experienced physicians to build trust and sustain clinical autonomy during AI integration [28,54].

The present study demonstrated that a sizeable proportion of PHC physicians responded to multiple items as needed or highly needed for the implementation of AI-based adaptive learning, particularly in preventive care and screening, mental health assessment, and management. These findings indicate that, even though the physicians had positive perceptions, they may have limited practical experience and related knowledge gaps leading to higher training needs [36,58]. Therefore, structured training is required to build confidence and reduce anxieties about adopting new technologies. The higher reported learning needs among non-Saudi physicians should be interpreted cautiously, as they may reflect differences in prior training exposure, familiarity with local PHC systems, and institutional orientation or educational support rather than nationality itself. Within a readiness framework, higher reported learning needs may reflect not only educational gaps, but also greater awareness of the support required for effective engagement with AI-based adaptive learning. In this sense, the higher learning needs reported by non-Saudi physicians and more experienced physicians may partly indicate greater self-awareness and readiness for targeted training. In the Saudi health context, language (a common barrier for expatriates) is noted as one of the important practical implementation obstacles [56,59]. From a policy perspective, PHC leaders can translate these needs into target-oriented measures that are aligned with PHC service priorities.

In the present study, we found that perceptions and needs are positively correlated. In contrast, both domains are negatively correlated with the barriers. Possible reasons include that physicians with positive perceptions may seek more opportunities and training needs. Some conceptual overlap between the perception and barrier domains should be acknowledged, especially for items related to digital self-efficacy and reservations toward AI-based learning. This may partly explain the moderate inverse correlation between the two domains and further supports an integrated readiness framework. The observed higher perceived barriers reflect lower implementation readiness, reduced motivation to pursue further upskilling, and fewer training needs. These associations can be further explained by previous studies that demonstrated that physicians’ acceptance is closely related to its usefulness, while the barriers undermine adoption [39,60]. According to a recent study (2025) by Lee et al., these findings may also be interpreted through technology-adoption perspectives such as TAM and UTAUT, in which perceived usefulness, ease of use, trust, and facilitating conditions influence willingness to adopt new technologies [39]. Implementation may also be constrained by practical barriers such as limited digital infrastructure, poor interoperability, data privacy concerns, and added workload burden in busy primary care settings. These system-level issues may affect readiness even when perceptions are generally favorable.

Our findings are broadly consistent with the global literature on AI adoption in healthcare, which suggests that uptake is influenced by perceived usefulness, digital literacy, training, and organizational support. Similar concerns about complexity, limited familiarity, and implementation barriers have also been reported internationally, supporting the view that readiness for AI-based adaptive learning is shaped by both individual and system-level factors [28,29,61].

The present study has several strengths. Firstly, this study was conducted in northern Saudi Arabia, a setting that is relatively underexplored in AI and digital health research in primary care. Secondly, the study included primary care physicians of all cadres and both Saudi and non-Saudi nationals. Moreover, the present study used a validated data collection tool. Finally, we performed correlation analysis and adjusted regression models to explore associated factors. However, some limitations must be acknowledged. Firstly, the study design used (cross-sectional) cannot establish the causal association. Next, even though the authors attempted to sample all representative populations, the use of convenience sampling limits the generalizability of the findings. This study was limited to two provinces. Therefore, caution must be exercised when generalizing to other regions of Saudi Arabia, as infrastructure and training needs vary. Although median-based dichotomization is commonly used in survey-based healthcare research, it may have led to some loss of information from the original continuous scores. In addition, the sample size was estimated for cross-sectional descriptive purposes rather than specifically for multivariable logistic regression, although the sample achieved was considered adequate for the included covariates. Moreover, the use of convenience sampling may have introduced selection bias and limited the representativeness of the study sample. Finally, bias due to self-reported study, such as recall bias, exaggerated responses, and possible social desirability bias in perceptions toward AI, cannot be ignored.

## 5. Conclusions

Primary care physicians working in PHCs in northern Saudi Arabia generally demonstrated favorable perceptions of AI-based adaptive learning, alongside measurable implementation barriers and substantial learning needs. We demonstrated several key associated factors, namely, physicians aged 40 years or older and non-Saudi nationals, across three domains. Policy efforts should include structured AI training modules, improved digital infrastructure and institutional support, and gradual integration of AI-based adaptive learning into CME programs. Furthermore, arrangements should be made for hybrid approaches (traditional and online) to better meet their learning needs, and these needs should be translated into target-oriented measures aligned with PHC service priorities. These measures could strengthen physicians’ readiness for AI-based adaptive learning in primary care settings. Furthermore, a mixed-methods survey is suggested to explore the qualitative views of AI-based adaptive learning among primary care physicians.

## Figures and Tables

**Table 1:** Sociodemographic and professional characteristics of primary care physicians (n = 285).

Variables	Frequency	Proportion (%)
Age (years) (Mean ± SD)	35.29 ± 7.79	
Age group (years)		
Up to 30	100	35.1
31 to 40	119	41.8
More than 40	66	23.2
Gender		
Male	131	46.0
Female	154	54.0
Nationality		
Saudi	206	72.3
Non-Saudi	79	27.7
Highest qualification		
MBBS	83	29.1
MD or equivalent	61	21.4
Saudi Board	141	49.5
Current position		
Resident	102	35.8
Specialist	97	34.0
Consultant	86	30.2
Work experience (years)		
Up to 5	100	35.1
5 to 10	108	37.9
More than 10	77	27.0
Use of digital tools for clinical learning		
No	62	21.8
Yes	223	78.2

**Table 2:** Current continuing medical education (CME) practices of the participants (n = 285).

Characteristics	Frequency	Proportion (%)
Participation in CME		
Monthly	79	27.7
2 to 3 months	111	38.9
Rarely/never	95	33.3
Participation in CME activities related to digital health, AI or e-learning		
No	75	26.3
Yes	210	73.7
Preferred CME method *		
In-person workshops/lectures (traditional)	198	69.5
Online (Webinars)/Virtua	252	88.4
Self-paced e-learning modules	186	65.3
Mobile-based app	206	72.3
Printed materials	157	55.1
Most common motivations to participate in CME		
License renewal requirements	41	14.4
Professional development	115	40.4
Institutional encouragement	26	9.1
Peer/Mentor influence	44	15.4
Interest in the topic	59	20.7

* More than once preferred choices.

**Table 3:** Descriptive statistics of study domains (Perceptions, Barriers, Needs) (n = 285).

Domains	Range	Mean ± SD	Median (IQR)	Min–Max
Perceptions	25	24.47 ± 4.50	24 (6)	10–35
Barriers	27	21.61 ± 5.81	21 (8)	8–35
Needs	32	29.11 ± 7.68	31 (11)	8–40

**Table 4:** Correlation matrix between perceptions, barriers, and needs domain scores (n = 285).

Domains	Spearman’s Rho (ρ)	*p*-Value
Perceptions vs. Barriers	−0.490	<0.001
Perceptions vs. Needs	0.539	<0.001
Needs vs. Barriers	−0.402	<0.001

**Table 5:** Factors associated with perceptions toward AI-based adaptive learning among primary care physicians.

Variable	Total	Perceptions		Adjusted Odds Ratio (AOR) (95% CI)	*p*-Value
		Low (n = 157)	High (n = 128)		
Age group (years)					
Up to 30	100	40	60	Ref	
31 to 40	119	65	54	1.13 (0.75–2.06)	0.155
More than 40	66	52	14	2.14 (1.32–3.08)	0.019
Gender					
Male	131	78	53	Ref	
Female	154	79	75	1.35 (0.78–2.35)	0.282
Nationality					
Saudi	206	95	111	Ref	
Non-Saudi	79	62	17	2.67 (1.24–4.19)	0.003
Highest qualification					
MBBS	83	31	52	Ref	
MD or equivalent	61	44	17	1.81 (1.15–3.19)	0.023
Saudi Board	141	82	59	1.54 (0.93–2.23)	0.141
Current position					
Resident	102	45	57	Ref	
Specialist	97	58	39	2.71 (0.83–4.03)	0.118
Consultant	86	54	32	1.53 (0.69–3.35)	0.476
Work experience (years)					
Up to 5	100	30	70	Ref	
5 to 10	108	67	41	2.50 (0.67–4.55)	0.187
More than 10	77	60	17	2.92 (1.75–4.14)	0.001
Use of digital tools for clinical learning					
No	62	42	20	Ref	
Yes	223	115	108	0.72 (0.61–0.93)	0.038

**Table 6:** Factors associated with perceived barriers to adopting AI-based adaptive learning (n = 285).

Variable	Total	Barriers		AOR (95% CI)	*p*-Value
		High (n = 121)	Low (n = 164)		
Age group (years)					
Up to 30	100	31	69	Ref	
31 to 40	119	48	71	0.86 (0.54–2.07)	0.715
More than 40	66	42	24	2.59 (1.37–3.74)	0.031
Gender					
Male	131	55	76	Ref	
Female	154	66	88	1.21 (0.72–2.03)	0.471
Nationality					
Saudi	206	76	130	Ref	
Non-Saudi	79	45	34	0.93 (0.64–1.98)	0.857
Highest qualification					
MBBS	83	26	57	Ref	
MD or equivalent	61	35	26	0.78 (0.41–1.76)	0.092
Saudi Board	141	60	81	2.14 (0.91–3.42)	0.209
Current position					
Resident	102	37	65	Ref	
Specialist	97	42	55	0.97 (0.66–2.61)	0.056
Consultant	86	42	44	0.80 (0.64–1.59)	0.248
Work experience (years)					
Up to 5	100	27	73	Ref	
5 to 10	108	45	63	0.69 (0.48–2.67)	0.382
More than 10	77	49	28	1.56 (1.19–2.86)	0.020
Use of digital tools for clinical learning					
No	62	36	26	Ref	
Yes	223	85	138	0.54 (0.38–1.27)	0.074

**Table 7:** Factors associated with learning needs for AI-based adaptive learning (n = 285).

Variable	Total	Learning Needs		AOR (95% CI)	*p*-Value
		High	Low		
Age group (years)					
Up to 30	100	37	63	Ref	
31 to 40	119	69	50	0.68 (0.49–1.64)	0.381
More than 40	66	46	20	1.64 (0.86–2.47)	0.521
Gender					
Male	131	72	59	Ref	
Female	154	80	74	1.11 (0.63–1.85)	0.694
Nationality					
Saudi	206	97	109	Ref	
Non-Saudi	79	55	24	1.65 (1.06–2.38)	0.017
Highest qualification					
MBBS	83	35	48	Ref	
MD or equivalent	61	32	29	1.70 (1.29–2.86)	0.008
Saudi Board	141	85	56	1.52 (0.84–3.46)	0.536
Current position					
Resident	102	44	58	Ref	
Specialist	97	61	36	1.08 (0.68–2.62)	0.714
Consultant	86	47	39	1.23 (0.70–3.19)	0.154
Work experience (years)					
Up to 5	100	34	66	Ref	
5 to 10	108	65	43	1.77 (1.14–2.46)	0.035
More than 10	77	53	24	2.65 (1.38–4.01)	0.007
Use of digital tools for clinical learning					
No	62	37	25	Ref	
Yes	223	115	108	0.97 (0.59–1.96)	0.608

## Data Availability

The original contributions presented in this study are included in the Supplementary Materials. Further inquiries can be directed to the corresponding author.

## Supplementary Materials

The following supporting information can be downloaded at: https://www.mdpi.com/article/10.3390/healthcare14070865/s1, Supplementary File S1: Exploratory factor analysis and factor loadings of the study questionnaire domains. Supplementary Table S1: Item-level responses for perceptions toward AI-based adaptive learning (7 items) among primary care physicians (n = 285). Supplementary Table S2: Item-level responses for barriers to adopting AI-based adaptive learning (7 items) among primary care physicians (n = 285). Supplementary Table S3: Item-level learning needs for AI-based adaptive learning implementation (8 items) among primary care physicians (n = 285). Supplementary Figure S1: Participant recruitment flow diagram.
