# Supplementary material for: Assessing Primary Care Physicians’ Readiness for AI-Based Adaptive Learning: Perceptions, Barriers, and Learning Needs in Northern Saudi Arabia
Source: Healthcare (Basel). 2026 Mar 27;14(7):865. doi: 10.3390/healthcare14070865 (PMC13073583; doi:10.3390/healthcare14070865)
Supplement: Supplementary file 1 [file healthcare-14-00865-s001.zip › EFA.pdf]

Supplementary file S1: Exploratory factor analysis and factor loadings of the study questionnaire domains

Items	Factor 1 (Perception)	Factor 2 (Barrier)	Factor 3 (Learning needs)
Perception item 1	0.641		
Perception item 2	0.713		
Perception item 3	0.684		
Perception item 4	0.605		
Perception item 5	0.759		
Perception item 6	0.633		
Perception item 7	0.720		
Barrier item 1		0.679	
Barrier item 2		0.607	
Barrier item 3		0.772	
Barrier item 4		0.652	
Barrier item 5		0.814	
Barrier item 6		0.744	
Barrier item 7		0.616	
Learning need item 1			0.663
Learning need item 2			0.783
Learning need item 3			0.602
Learning need item 4			0.640
Learning need item 5			0.802
Learning need item 6			0.616
Learning need item 7			0.693
Learning need item 8			0.757
