# Supplementary material for: Assessing Primary Care Physicians’ Readiness for AI-Based Adaptive Learning: Perceptions, Barriers, and Learning Needs in Northern Saudi Arabia
Source: Healthcare (Basel). 2026 Mar 27;14(7):865. doi: 10.3390/healthcare14070865 (PMC13073583; doi:10.3390/healthcare14070865)
Supplement: Supplementary file 1 [file healthcare-14-00865-s001.zip › healthcare-4172562-supplementary_revised.pdf]

Supplementary Table S1. Item-level responses for perceptions toward AI-based adaptive learning (7 items) among primary care physicians (n=285)

Items	Strongly disagree n (%)	Disagree n (%)	Neutral n (%)	Agree n (%)	Strongly agree n (%)
I believe adaptive learning can provide more targeted educational content than traditional CME.	12 (4.2)	6 (2.1)	25 (8.8)	106 (37.2)	136 (47.7)
I believe AI-based learning tools can improve the effectiveness of CME for primary care.	11 (3.9)	6 (2.1)	26 (9.1)	121 (42.5)	121 (42.5)
I feel confident in my ability to use AI-based learning platforms without assistance.	12 (4.2)	19 (6.7)	54 (18.9)	130 (45.6)	70 (24.6)
AI-based learning systems are too complex for most doctors to use effectively *.	21 (7.4)	57 (20.0)	78 (27.4)	77 (27.0)	52 (18.2)
Personalized learning tools would help me better identify and address my clinical knowledge gaps.	2 (0.7)	16 (5.6)	41 (14.4)	110 (38.6)	116 (40.7)
I am concerned that AI-based platforms might replace human educators *.	21 (7.4)	40 (14.0)	65 (22.8)	104 (36.5)	55 (19.3)
I prefer traditional CME methods and have no interest in digital learning platforms *.	48 (16.8)	72 (25.3)	58 (20.4)	76 (26.7)	31 (10.9)

* Reverse scored

Supplementary Table S2. Item-level responses for barriers to adopting AI-based adaptive learning (7 items) among primary care physicians (n=285)

Items	Strongly disagree n (%)	Disagree n (%)	Neutral n (%)	Agree n (%)	Strongly agree n (%)
I have limited time to explore or engage with new learning technologies.	22 (7.7)	90 (31.6)	85 (29.8)	66 (23.2)	22 (7.7)
My institution does not currently support or promote AI-based learning platforms.	15 (5.3)	93 (32.6)	94 (33.0)	61 (21.4)	22 (7.7)
I am not confident in using digital tools for education.	23(8.1)	97 (34.0)	82 (28.8)	63 (22.1)	20 (7.0)
AI-based learning is too impersonal and lacks human interaction.	15 (5.3)	114 (40.0)	64 (22.5)	70 (24.6)	22 (7.7)
I have never received formal training or orientation on how to use adaptive learning platforms.	12 (4.2)	89 (31.2)	70 (24.6)	88 (30.9)	26 (9.1)

I am concerned that over-reliance on AI tools might reduce my critical thinking over time.	14 (4.9)	71(24.9)	63(22.1)	105 (36.8)	32 (11.2)
Technical issues (e.g., internet, software glitches) often limit my ability to use online platforms effectively.	18 (6.3)	76 (26.7)	65 (22.8)	95 (33.3)	31 (10.9)

Supplementary Table S3. Item-level learning needs for AI-based adaptive learning implementation (8 items) among primary care physicians (n=285)

Items	Not needed n (%)	Slightly needed n (%)	Moderately needed n (%)	Needed n (%)	Highly needed n (%)
Clinical decision-making and diagnostic reasoning in complex primary care cases	12 (4.2)	36 (12.6)	89 (31.2)	93 (32.6)	55 (19.3)
Preventive care and screening guidelines across age groups	14 (4.9)	35 (12.3)	54 (18.9)	117 (41.1)	65 (22.8)
Mental health assessment and management in primary care	13 (4.6)	28 (9.8)	66 (23.2)	106 (37.2)	72 (25.3)
Emergency and urgent care protocols in outpatient settings	13 (4.6)	36 (12.6)	65 (22.8)	111 (38.9)	60 (21.1)
Communication skills (e.g., patient counselling, breaking bad news)	17 (6.0)	34 (11.9)	61 (21.4)	105 (36.8)	68 (23.9)
Use of digital health tools and telemedicine in daily practice	13 (4.6)	25 (8.8)	65 (22.8)	105 (36.8)	77 (27.0)
Interpretation of evidence-based guidelines and research for practice	13 (4.6)	33 (11.6)	63 (22.1)	103 (36.1)	73 (25.6)
Time management and handling clinical workload effectively	18 (6.3)	29 (10.2)	58 (20.4)	105 (36.8)	75 (26.3)
