# Supplementary figures and images for: Assessing Primary Care Physicians’ Readiness for AI-Based Adaptive Learning: Perceptions, Barriers, and Learning Needs in Northern Saudi Arabia
Source: Healthcare (Basel). 2026 Mar 27;14(7):865. doi: 10.3390/healthcare14070865 (PMC13073583; doi:10.3390/healthcare14070865)

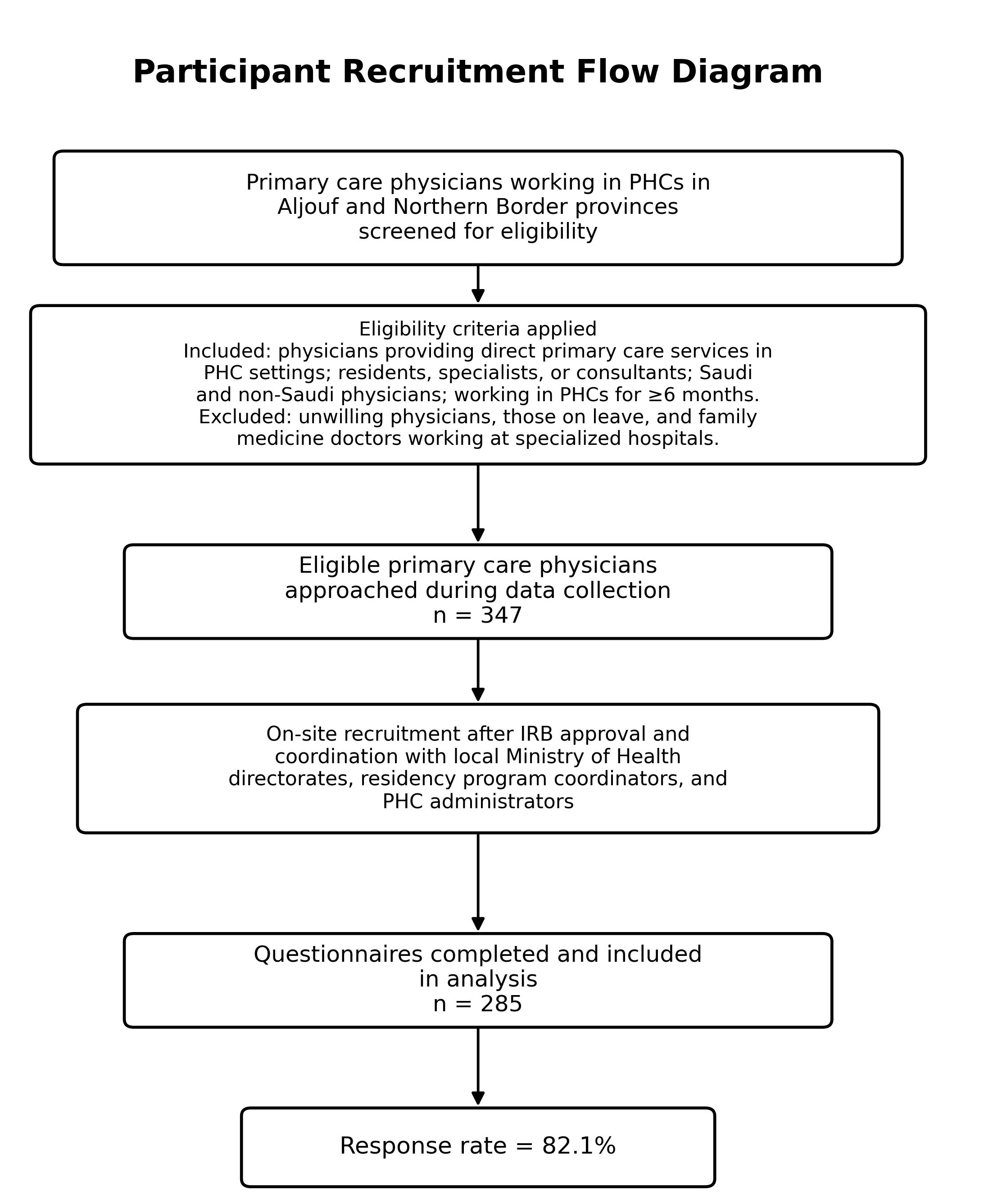

Supplement: Supplementary file 1 [file healthcare-14-00865-s001.zip › Figure S1 - Participant recruitment flow diagram.png]
